# Supplementary material for: ADAR1 averts fatal type I interferon induction by ZBP1
Source: Nature. 2022 Jul 20;607(7920):776–83. doi: 10.1038/s41586-022-04878-9 (PMC9329096; doi:10.1038/s41586-022-04878-9)
Supplement: Supplementary file 1 — Uncropped gels for immunoblots presented in the manuscript. [file 41586_2022_4878_MOESM1_ESM.pdf]

nature portfolio

https://doi.org/10.1038/s41586-022-04878-9

Supplementary information

ADAR1 averts fatal type I interferon induction by ZBP1

In the format provided by the authors and unedited

nature portfolio

https://doi.org/10.1038/s41586-022-04878-9

Supplementary information

ADAR1 averts fatal type I interferon induction by ZBP1

In the format provided by the authors and unedited

Figure 1f

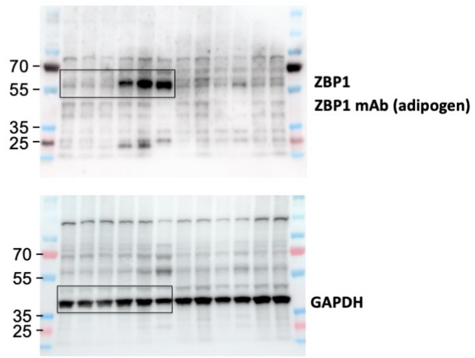

Loading control from ZBP1 blot

ED Figure 6i

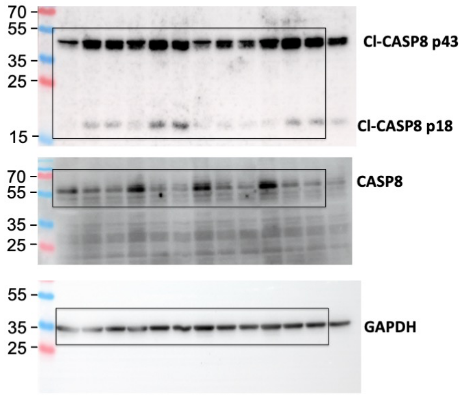

Loading control from Cl-CASP8 and CASP8 blots

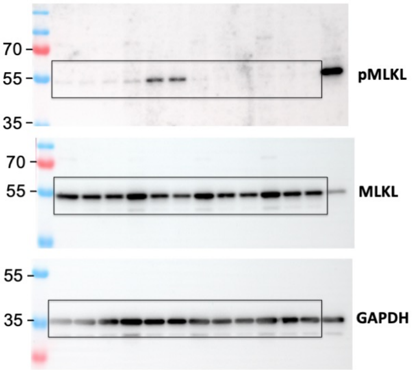

Loading control from pMLKL and MLKL blots

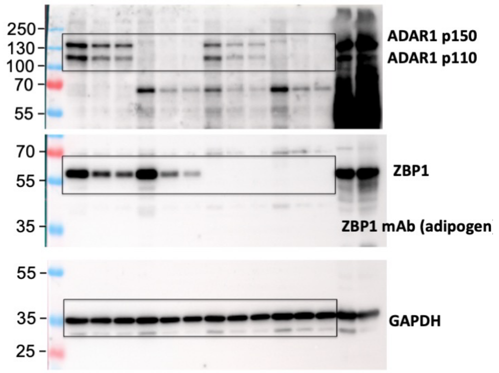

Loading control from ADAR1 and ZBP1 blots

ED Figure 6f

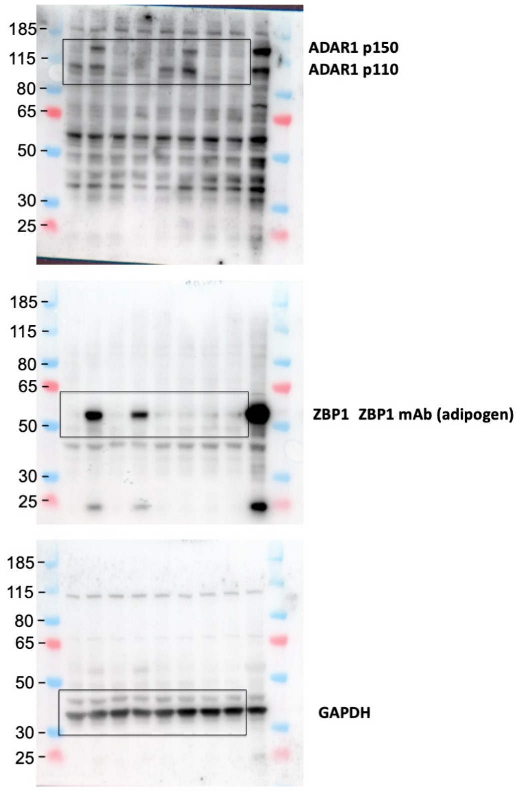

Loading control from ADAR1 and ZBP1 blots

ED Figure 7b

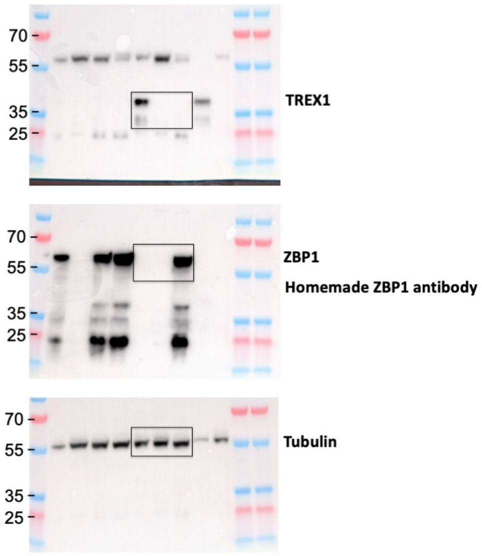

Loading control from TREX1 and ZBP1 blots

ED Figure 7c

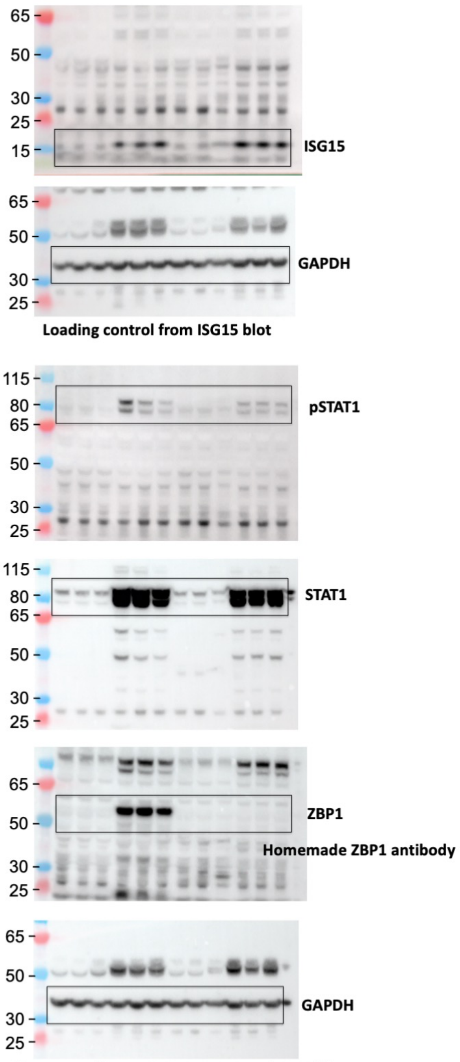

Loading control from pSTAT1, STAT1 and ZBP1 blots
