# Supplementary material for: ADAR1 averts fatal type I interferon induction by ZBP1
Source: Nature. 2022 Jul 20;607(7920):776–83. doi: 10.1038/s41586-022-04878-9 (PMC9329096; doi:10.1038/s41586-022-04878-9)
Supplement: Supplementary file 2 — Reporting Summary [file 41586_2022_4878_MOESM2_ESM.pdf]

nature portfolio

Corresponding author(s): Manolis Pasparakis

Last updated by author(s): Apr 27, 2022

Reporting Summary

Nature Portfolio wishes to improve the reproducibility of the work that we publish. This form provides structure for consistency and transparency in reporting. For further information on Nature Portfolio policies, see our Editorial Policies and the Editorial Policy Checklist.

Statistics

For all statistical analyses, confirm that the following items are present in the figure legend, table legend, main text, or Methods section.

n/a	Confirmed	
☐	☒	The exact sample size (n) for each experimental group/condition, given as a discrete number and unit of measurement
☐	☒	A statement on whether measurements were taken from distinct samples or whether the same sample was measured repeatedly
☐	☒	The statistical test(s) used AND whether they are one- or two-sided<br>*Only common tests should be described solely by name; describe more complex techniques in the Methods section.*
☒	☐	A description of all covariates tested
☐	☒	A description of any assumptions or corrections, such as tests of normality and adjustment for multiple comparisons
☒	☐	A full description of the statistical parameters including central tendency (e.g. means) or other basic estimates (e.g. regression coefficient) AND variation (e.g. standard deviation) or associated estimates of uncertainty (e.g. confidence intervals)
☒	☐	For null hypothesis testing, the test statistic (e.g. F, t, r) with confidence intervals, effect sizes, degrees of freedom and P value noted<br>*Give P values as exact values whenever suitable.*
☒	☐	For Bayesian analysis, information on the choice of priors and Markov chain Monte Carlo settings
☒	☐	For hierarchical and complex designs, identification of the appropriate level for tests and full reporting of outcomes
☒	☐	Estimates of effect sizes (e.g. Cohen's d, Pearson's r), indicating how they were calculated

Our web collection on statistics for biologists contains articles on many of the points above.

Software and code

Policy information about availability of computer code

Data collection	Cell death assay data was collected by using IncuCyte S3 2018B (sartorius). QPCR data was collected by using QuantStudio 12K Flex Software (applied biosystems). Imaging data was collected by using NanoZoomer S360 Digital slide scanner C13220-01, Zeiss Axio Imager microscope and Ventana DP 200 slide scanner. Western blot data was collected by using Vilber FUSION SOLO X. Whole blood analysis data of the mice was collected by using Abacus Junior vet.
Data analysis	Statistical analysis was performed with GraphPad Prism V6. Cell death assay data was analysed by using IncuCyte S3 2018B(sartorius). Pathway analyses were performed using g:Profiler (https://biit.cs.ut.ee/gprofiler). kmers were converted into complementary kmers using shell commands and Python code. Pictures were analyzed and processed using the Omero software package (openmicroscopy.org) and the NDP.view2 Viewing software (Hamamatsu). Nuclei segmentation of individual tufts of kidney sections was performed by applying the built-in nucleus segmentation tool (QuPath). The quality of the resulting RNA-seq data was assessed using FastQC v0.11.8 (bioinformatics.babraham.ac.uk/projects/fastqc/). DESeq2 v1.22.1 within R was used for the read count normalisation and downstream differential expression analysis and visualisation was conducted within Qlucore Omics Explorer v3.3 (Qlucore, Lund, Sweden). A-to-I editing of RNA-seq data was assessed using JACUSA2.

For manuscripts utilizing custom algorithms or software that are central to the research but not yet described in published literature, software must be made available to editors and reviewers. We strongly encourage code deposition in a community repository (e.g. GitHub). See the Nature Portfolio guidelines for submitting code & software for further information.

Data

Policy information about availability of data

All manuscripts must include a data availability statement. This statement should provide the following information, where applicable:

- Accession codes, unique identifiers, or web links for publicly available datasets
- A description of any restrictions on data availability
- For clinical datasets or third party data, please ensure that the statement adheres to our policy

RNA-seq data have been deposited in the ArrayExpress database at EMBL-EBI (www.ebi.ac.uk/arrayexpress) under accession number E-MTAB-10953. All data supporting the findings of this study are available from the corresponding author on reasonable request.

Field-specific reporting

Please select the one below that is the best fit for your research. If you are not sure, read the appropriate sections before making your selection.

☒ Life sciences ☐ Behavioural & social sciences ☐ Ecological, evolutionary & environmental sciences

For a reference copy of the document with all sections, see nature.com/documents/nr-reporting-summary-flat.pdf

Life sciences study design

All studies must disclose on these points even when the disclosure is negative.

Sample size	Sample size was determined empirically and was based on our previous work using RIPK1mR/mR, RIPK1E-KO, FADDIEC-KO mice. We aimed for a number of at least 3 animals per group to allow basic statistical analysis while using a justifiable number of mutant mice. Based on previous experience from similar studies, in vitro experiments with cultured cells for cell death assay were performed at least 3 times (biological replicates including 3 technical replicates for each experiment) to confirm reproducibility.
Data exclusions	No data was excluded from the analysis.
Replication	Whenever possible, readouts were performed with at least 3 animals of a given genotype. For in vitro studies we independently replicated all experiments at least 3 times (biological replicates) for cell death assay (including 3 technical replicates for all the experiments) and at least two times (biological replicates) for the immunoblotting analysis. All attempts at replication were successful.
Randomization	No specific method of randomization had been used to select animals. We compared groups of mice with different genotypes to assess the effect of specific genetic mutations in the phenotype. Group allocation was thus determined by the genotype of the mice.
Blinding	No blinding was done during and group generation as the group allocation was determined by the genotype of the mice. Histological evaluation of different tissues was performed blindly.

Reporting for specific materials, systems and methods

We require information from authors about some types of materials, experimental systems and methods used in many studies. Here, indicate whether each material, system or method listed is relevant to your study. If you are not sure if a list item applies to your research, read the appropriate section before selecting a response.

Materials & experimental systems

n/a	Involved in the study	
☐	☒	Antibodies
☐	☒	Eukaryotic cell lines
☒	☐	Palaeontology and archaeology
☐	☒	Animals and other organisms
☒	☐	Human research participants
☒	☐	Clinical data
☒	☐	Dual use research of concern

Methods

n/a	Involved in the study	
☒	☐	ChIP-seq
☒	☐	Flow cytometry
☒	☐	MRI-based neuroimaging

Antibodies

Antibodies used	1 monoclonal rat anti-CD45 (30-F11), Cat. No. 14-0451-85, eBioscience, Dilution 1:1000 for IHC, Lot. No. E03735-1631;<br>2 monoclonal rabbit anti-Phospho-MLKL (Ser345) (D6E3G), Cat. No. 37333, Cell Signaling Technology, Dilution 1:1000 for WB, Lot. No. 2;<br>3 monoclonal rat anti-MLKL (3H1), Cat. No. MABC604, Millipore, Dilution 1:1000 for WB, Lot. No. 3256617;<br>4 monoclonal mouse anti-ZBP1 (Zippy-1), Cat. No. AG-20B-0010, Adipogen, Dilution 1:1000 for WB, Lot. No. A28231605;

5 monoclonal mouse anti-GAPDH (1D4), Cat. No. NB300-221, NovusBiologicals, Dilution 1:1000 for WB, Lot. No. 082219;
6 monoclonal rabbit anti-Cleaved Caspase-8 (Asp387) (D5B2), Cat. No. 8592, Cell Signaling Technology, Dilution 1:1000 for WB, Lot. No. 3;
7 monoclonal rabbit anti-Caspase-8 (D35G2), Cat. No. 4790, Cell Signaling Technology, Dilution 1:1000 for WB, Lot. No. 2;
8 monoclonal rat anti-F4/80, Cat. No. MCA497, clone A3-1, AbD Serotec, dilution for IHC 1:75;
9 donkey anti-rabbit IgG – HRP, Cat. No. NA934, GE Healthcare, Dilution 1:4000 for WB, Lot. No. 17041907;
10 sheep anti-mouse IgG – HRP, Cat. No. NA931, GE Healthcare, Dilution 1:4000 for WB, Lot. No. 17028693;
11 goat anti-rat IgG – HRP, Cat. No. 112-035-003, Jackson Immuno Research, Dilution 1:4000 for WB, Lot. No. 144357;
12 goat anti-rat IgG – Biotin-SP, Cat. No. 112-065-003, Jackson Immuno Research, Dilution 1:1000 for IHC, Lot. No.112632 ;
13 monoclonal rabbit anti-Phospho-STAT1 (tyr701) (58D6), Cat. No. 9167, Cell Signaling Technology, Dilution 1:1000 for WB, Lot. No. 25;
14 polyclonal rabbit anti-STAT1, Cat. No. 9172, Cell Signaling Technology, Dilution 1:1000 for WB, Lot. No. 25;
15 polyclonal rabbit anti-ISG15, Cat. No. 2743, Cell Signaling Technology, Dilution 1:1000 for WB, Lot. No. 3;
16 monoclonal mouse anti-ADAR1 (15.8.6), Cat. No. sc-73408, Santa Cruz Biotechnology, Dilution 1:500 for WB, Lot. No. H0119;
17 monoclonal rat anti-CD3 (CD3-12), Cat. No. MCA1477, BIO-RAD, Dilution 1:100 for IHC, Lot. No. 149500D or I55872;
18 polyclonal rabbit anti-Cleaved Caspase-3 (Asp175) (D5B2), Cat. No. 9661, Cell Signaling Technology, Dilution 1:500 for IHC, Lot. No. 47;
19 monoclonal mouse anti-TREX1 (C-11), Cat. No. sc-133112, Santa Cruz Biotechnology, Dilution 1:1000 for WB, Lot. No. E1618;
20 monoclonal mouse anti- α-tubulin (B-5-1-2), Cat. No. T6074, Sigma, Dilution 1:5000 for WB, Lot. No. 046M4763V;
21 polyclonal goat anti-rabbit IgG (H+L), Cat. No. B-2770, Invitrogen, Dilution 1:1000 for IHC, Lot. 2300198
22 monoclonal rabbit anti-Iba1(EPR16588), Cat. No. ab178846, Abcam, Dilution 1:300 for IHC, Lot. No. GR3335980 3;
23 monoclonal rat anti-Mac-3 (M3/84), Cat. No. 550292, BD Biosciences, Dilution 1:200 for IHC, Lot. No. 1076190;
24 monoclonal rat anti-B220 (RA3-6B2), Cat. No. 553084, BD Biosciences, Dilution 1:200 for IHC, Lot. No. 0072031;
25 goat anti-rabbit IgG (H+L), Cat. No. 4050-08, Southern Biotech, dilution 1:200, Lot. No. I4114-N395X;
26 polyclonal rabbit anti-ZBP1 serum (custom-made by Eurogentec), Dilution 1:1000 for WB.

Validation

Validation data for all the commercial antibodies are available on vendor websites.
Custom-made ZBP1 antibody is validated for WB using ZBP1 KO cells in this manuscript and ZBP1 KO skin lysates in Lin, J., Kumari, S., Kim, C. et al. RIPK1 counteracts ZBP1-mediated necroptosis to inhibit inflammation. Nature 540, 124–128 (2016).

Eukaryotic cell lines

Policy information about cell lines

Cell line source(s)

Mouse embryonic fibroblasts derived from the mice.

Authentication

We used primary mouse embryonic fibroblasts derived from the mice.

Mycoplasma contamination

We do not screen primary cell cultures for Mycoplasma.

Commonly misidentified lines
(See ICLAC register)

No ISLAC cell lines were used in this study

Animals and other organisms

Policy information about studies involving animals; ARRIVE guidelines recommended for reporting animal research

Laboratory animals

C57BL/6 male and female mice, between P0 and 53 weeks of age, were used in these studies.

Wild animals

This study did not involve wild animals.

Field-collected samples

This study did not involve samples collected from the field.

Ethics oversight

All animal procedures were conducted in accordance with national and institutional guidelines and protocols were approved by the responsible local authorities in Cologne (Landesamt für Natur, Umwelt und Verbraucherschutz Nordrhein-Westfalen, Germany).

Note that full information on the approval of the study protocol must also be provided in the manuscript.
